# Epistemic Emotions and Observations Are Intertwined in Scientific Sensemaking: A Study among Upper Secondary Physics Students

**DOI:** 10.1007/s10763-022-10310-5

**Published:** 2022-09-05

**Authors:** Elisa Vilhunen, Mei-Hung Chiu, Katariina Salmela-Aro, Jari Lavonen, Kalle Juuti

**Affiliations:** 1 Faculty of Educational Sciences, University of Helsinki, P.O 9 (Siltavuorenpenger 5A), 00014 Helsinki, Finland; 2 Graduate Institute of Science Education, National Taiwan Normal University, Taipei, Taiwan

**Keywords:** Epistemic emotions, Observation, Scientific sensemaking, Upper secondary school

## Abstract

This study contributes to the understanding of the relationship between emotions and development of scientific understanding by examining (1) how students perform in scientific sensemaking in the context of a three-cycle predict-observe-explain (POE) activity, (2) what kind of trajectories of situational epistemic emotions students show when making sense of the phenomenon, and (3) how students’ performance in sensemaking is related to their emotional trajectories. Data from 109 participant students were collected in six upper secondary physics classes. Students’ performance in sensemaking was evaluated based on their answers on POE items and categorised through qualitative content analysis. Situational epistemic emotions (surprise, curiosity, confusion, and boredom) were measured using a four-point Likert scale after each POE cycle. Latent class growth analysis was used to identify groups of students with distinctive emotional trajectories. The relationship between the performance in POE activity and emotional trajectories was explored by a chi-square test. The results indicate that students’ inability to make relevant observations is significantly related to experienced boredom. Furthermore, students who perform better in making sense of the phenomenon are more likely to experience surprise, curiosity, and confusion. This implies that engaging students to be curious when they observe and test predictions is an important mission for curriculum designers and teachers in practice. The findings underline the importance of epistemic emotions in educational settings and the complexity of the interplay between cognitive and affective factors in learning situations.

**Supplementary Information:**

The online version contains supplementary material available at 10.1007/s10763-022-10310-5.

## Introduction

Emotions and cognition are known to be intertwined in general (e.g. Fischer et al., 2014; Pekrun et al., 2018) and in the context of science teaching and learning (e.g. Davis & Bellocchi, 2018; Theobald & Brod, 2021; Vilhunen et al., 2022; Wickman et al., 2022). Acknowledging the role of emotions in science education can promote students’ interest, engagement, and learning outcomes (e.g. King et al., 2015; Schneider et al., 2020). Characteristic to science learning is making sense of scientific phenomena (e.g. Krajcik, 2015). Scientific sensemaking is often considered to be a rational, highly analytical practice, and the role of emotions or other affective factors in sensemaking has too often been neglected (e.g. Sinatra & Pintrich, 2003). Despite the large body of research on sensemaking practices, such as modelling or explaining (Odden & Russ, 2019b; Schwarz et al., 2009), and a general assumption that emotions influence scientific reasoning (Fischer et al., 2014), there is still little scientific understanding of how sensemaking and emotions are intertwined in authentic science learning situations. Generating understandings about emotional experiences in scientific sensemaking can enhance students’ science learning, interest, and engagement. This study advances the recent investigations by exploring students’ scientific sensemaking and their situational emotional experiences within a three-cycle predict-observe-explain activity, in the context of Finnish upper secondary school physics. This study contributes to the understanding of the relationship between emotions and learning, by providing novel perspectives for science educators and researchers on how students’ emotions and their development of scientific understanding are intertwined in science learning.

### Making Sense of Scientific Phenomena

Recently, making sense of phenomena has been of growing interest in the field of science education (e.g. Ding et al., 2021; Krajcik, 2015; Kubsch et al., 2020; National Research Council, 2012). The ability to make sense of phenomena is essential for developing scientific understanding and advancing critical thinking. Odden and Russ (2019a, p.187) define scientific sensemaking as “the process of building an explanation to resolve a perceived gap or conflict in knowledge”. In line with this, scientific sensemaking can also be considered a hypernym for several scientific practices, such as modelling, reasoning, explaining, or argumenting (National Research Council, 2012). Making sense of scientific phenomena is a pertinent part of any type of inquiry learning, and thus, many instructional approaches aim at sensemaking. For example, project-based learning (Krajcik & Shin, 2014), inquiry-based instruction (Minner et al., 2010), or modelling-based learning (Schwarz et al., 2009) are all grounded in making sense of phenomena. In the present study, the terms *phenomena* and *scientific phenomena* both refer to such natural phenomena that are explained (or explainable) through science.

Furthermore, scientific sensemaking is emphasised in the Framework for K-12 Science Education (National Research Council, 2012), as well as in the Finnish national curriculum (Finnish National Board of Education, 2016a), i.e. the curriculum followed in the context of the present research. Previous studies have indicated that a context and cognitive tools provided, such as graphical representations, can influence students’ sensemaking processes (e.g. Ding et al., 2021; Kubsch et al., 2020). In this study, a predicting-observing-explaining (POE) strategy (White & Gunstone, 1992) was employed to guide students to make sense of a scientific phenomenon.

In the *predicting* phase of the POE strategy, students are introduced to an inquiry activity. Students apply their initial model or preconception to make the prediction. White and Gunstone (1992) emphasise that predictions are not guesses; it is important to guide students to explain why they think that certain things would happen in the experiment (i.e. apply a model). In consecutive POE cycles, students may revise their prediction if they have recognised that their initial model did not predict the observation correctly or sufficiently.

In the *observing* phase, the experiment is carried out, the phenomenon is observed, and results are reported. Whereas everyday observations may appear just as looking at or noticing things, scientific observations should also have the aspect of describing or inferring things or phenomena (Eberbach & Crowley, 2009; Pinch, 1985). Thus, prior scientific knowledge, together with other cognitive and affective factors, can influence students’ observations (Hodson, 1996; Kohlhauf et al., 2011; Pinch, 1985). Furthermore, recent studies show that students may have difficulties in making scientific observations (Haigh et al., 2012; Remmen & Frøyland, 2020), indicating that the role of scaffolding is central in learning to observe scientific phenomena.

In the *explaining* phase, students explain their observations. They are guided to think how the previous explanation models should be revised or improved to explain the observations better. White and Gunstone (1992) emphasise that predicting engages pupils in deciding what knowledge is appropriate to apply in certain situations to come up with a rational explanation. Thus, the POE approach connects models as tools to explain observations, i.e. to find an answer to the questions and make sense of a phenomenon.

Several studies have employed POE strategy to probe students understanding on scientific phenomena and their critical thinking skills (e.g. Arsy et al., 2019; Hong et al., 2021). Furthermore, POE strategy has been utilised in studying students’ and preservice teachers’ emotions in science education (Bellocchi et al., 2014; Chiu et al., 2014, 2019; Liaw et al., 2020b).

### Epistemic Emotions

Emotions are typically defined as affective episodes that are caused by a certain stimulus or antecedent and have an object (Pekrun et al., 2018). In other words, emotions take place in situations and are caused by some external or internal factors. Thus, they are different from other affective variables, such as moods or attitudes, which are typically more stable and long lasting, and do not necessarily have such a clear stimulus nor an object (Shuman & Scherer, 2014). Emotions that have an object focus on knowledge or knowledge construction are further defined as epistemic emotions (Pekrun et al., 2018). Epistemic emotions, such as surprise, curiosity, confusion, or boredom, typically occur in situations of contradictory or incongruous information where new understandings are developed (Pekrun et al., 2017). Thus, epistemic emotions are especially interesting in terms of learning and scientific sensemaking. In general, positive emotions are typically linked to positive educational outcomes, whereas negative emotions are linked to negative outcomes (Darner, 2019; King et al., 2015; Liu et al., 2014; Muis et al., 2015; Vilhunen et al., 2022).

### Emotions in Scientific Inquiry

In learning situations, epistemic emotions often co-occur, correlate, or dynamically interact with each other constituting various emotional trajectories in learners (Bosch & D’Mello, 2017; D’Mello & Graesser, 2012). Making sense of phenomena and developing scientific understanding are cognitively demanding activities and thus can arouse variety of emotions in students. Recent research has recognised that students can have diverse affective experiences about scientific inquiry and sensemaking (Ding et al., 2021).

When encountering novel or contradictory information, surprise is usually the primary emotion (Chiu et al., 2014; D’Mello & Graesser, 2012; Theobald & Brod, 2021). Surprise has been considered important for learning since it is related to conceptual change (Chiu et al., 2014, 2019; Liaw, 2020), the knowledge revision process (Jacobson et al., 2021), and positive learning strategies, such as knowledge exploration (Vogl et al., 2020). Recent research has found that in science inquiry, generating predictions can induce surprise (Brod, 2021) and curiosity (Brod & Breitwieser, 2019) and thus engage students in scientific sensemaking (Theobald & Brod, 2021).

In cognitive disequilibrium, surprise is often followed by confusion or curiosity or both (D’Mello & Graesser, 2012). The positive effect of curiosity on learning is indisputable (Liaw et al., 2020; Pekrun et al., 2018; Schneider et al., 2020). Curiosity predicts positive learning strategies (Vogl et al., 2020), enhances memory (Gruber & Ranganath, 2019), and contributes to engagement in science inquiry (Wu et al., 2018). However, the role of negatively valenced confusion in learning and knowledge generation is more disputable. Some studies suggest that confusion relates to negative learning strategies (Bosch & D’Mello, 2017), hampers the scientific knowledge revision process (Jacobson et al., 2021), and can be considered a learning detractor (Schneider et al., 2016). On the other hand, some studies have shown that confusion can predict deep-processing learning strategies (Muis et al., 2015) and contribute positively to classroom dynamics and engagement in science inquiry (Watkins et al., 2018). Indeed, in the context of science learning, negatively valenced emotions have been considered to be pertinent, as being integral to learning and engagement and inherent in science (e.g. Jaber & Hammer, 2016; Radoff et al., 2019; Watkins et al., 2018). Cognitively demanding scientific activities with discrepant or perplexing information may arouse uncertainty and negative emotions, such as confusion or frustration, but on the other hand can lead to new realisations or achievements, which in turn may arouse for example curiosity or enjoyment (Radoff et al., 2019; Vilhunen et al., 2021). Also, previous research suggests that POE activities typically arouse relatively positive emotions in learners (Bellocchi et al., 2014).

If confusion is resolved and a cognitive equilibrium is achieved, students often feel enjoyment and engagement for learning (D’Mello & Graesser, 2012). However, if confusion persists and deepens, a student may feel stuck and experience a lack of control. For example, cognitive load in POE activity (Hong et al., 2017) or prolonged incongruence in scientific modelling (Han & Gutierez, 2021) has been found to induce negative emotions in students. Furthermore, Darner (2019) argues that negative emotions can even lead to denial of empirical evidence. Eventually, prolonged cognitive disequilibrium can lead to boredom and disengagement (Bosch & D’Mello, 2017; D’Mello & Graesser, 2012), which have been found to have detrimental effects on learning (Pekrun et al., 2014; Tze et al., 2016; Vilhunen et al., 2022). To conclude, in science learning, different trajectories of epistemic emotions can give rise to a complex interplay between cognitive and affective factors.

### The Current Study

In this study, we investigated the role of epistemic emotions in scientific sensemaking. This has been done by employing a computer-based three-cycle POE activity in Finnish upper secondary school physics and combining qualitative data on students’ predictions, observations, and explanations on a motion phenomenon with quantitative trajectories on students’ experiences of situational epistemic emotions. The research questions (RQs) are as follows:

- RQ1: How do students perform in making sense of the motion phenomenon within a three-cycle POE activity?
- RQ2: What kind of trajectories of situational epistemic emotions can be identified among students when making sense of the phenomenon within the POE activity?
- RQ3: How is student performance in scientific sensemaking related to their trajectories of situational epistemic emotions?

## Materials and Methods

### Context and Participants

The participants in the study were 113 students (56.6% female, ~ 16 years old) from six classes in a Finnish upper secondary school located in the Helsinki metropolitan area. A convenience sampling approach was used (Etikan et al., 2016). The teachers of the participating classes were recruited based on previous research collaboration. None of the researchers were teaching the students. The data were collected using a computer-based learning task, based on a three-cycle POE activity. The data collection took place during a first physics course in upper secondary school, just before the instruction on Newtonian mechanics (Finnish National Board of Education, 2016a). Thus, students’ previous knowledge on mechanics was based on lower secondary school physics. In lower secondary school, students inquire into movement with constant velocity and constant acceleration, such as free falling, and analyse reasons why velocity is sometimes changing and sometimes not changing (Finnish National Board of Education, 2016b).

The data were collected between autumn 2019 and spring 2021. In four of the participating classes, the data were gathered as part of the normal class work. However, in spring 2021, the students in two participating classes completed the computer-based learning task from homes as part of distance learning due to the COVID-19 pandemic.

Participation in the activity was part of the regular schoolwork. Participation in the research was voluntary, but informed consent was required from all participants whose data were used as part of the study. Furthermore, the data collection (i.e. the computer-based learning activity) was planned to be pedagogical from the students’ viewpoint and conducted in accordance with the curriculum. It took about 15 min for students to complete the learning task.

### Data Collection: the Computer-Based POE Activity

All the data on students’ scientific sensemaking and epistemic emotions were collected within a computer-based POE activity, in which students were introduced to three situations concerning the motion of a falling object. By posing relevant questions, students were guided through the three cycles of predicting, observing, and explaining to make sense of the phenomenon. A detailed description of the POE activity and the items used in it are given in a supplementary material. The activity was conducted in Finnish.

#### Situation 1

**Fig. 1:**
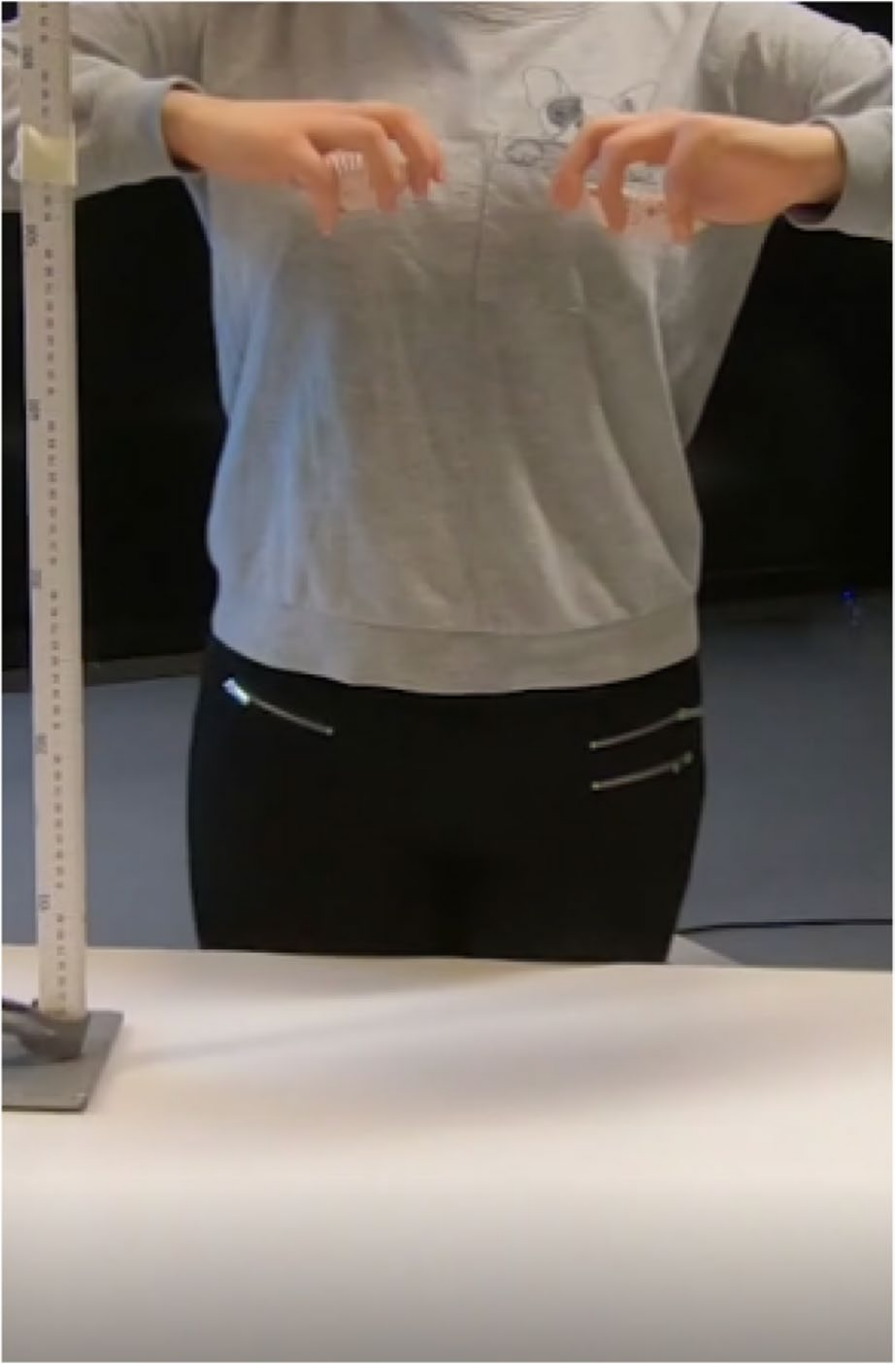
A still picture included in the computer-based POE activity

In the first situation, students were asked to compare the motion of 1 and 2 falling muffin cups. Students were first shown a still picture of the muffin cups (Fig. 1) and were asked to *predict* which pile of cups would hit the table first. Next, students were shown a video in which the muffin cups are dropped, and they were asked to describe their *observation* and *explain* it. Students were expected to observe that the heavier (i.e. 2 cups) falls faster. We expected this first situation to be intuitive for the students and to confirm their conceptions that heavier objects fall faster.

#### Situation 2

In the second situation, the mass of muffin cup piles was doubled, and 2 and 4 cups were dropped. In addition to the first situation, students were now asked to compare this situation to the earlier situation. Otherwise, the second situation followed the same POE procedure as the first situation. Students were expected to observe that the heavier pile (i.e. 4 cups) falls faster and that the time gap between the falling times of the two objects was smaller than in the previous situation.

#### Situation 3

In the third situation, the mass of the muffin cup piles was increased even more, and 16 and 32 cups were dropped. So, in each situation, the masses of the cup piles were *m* (object 1) versus 2* m* (object 2). Otherwise, the third situation was similar to the second one. In the third video, the cup piles hit the table seemingly at the same time, though a small time gap was observable if watched in slow motion. Thus, students were expected to make an observation that either the heavier (i.e. 32 cups) falls faster and the time gap between the falling times of the two objects was smaller than in the previous situation or that the cup piles hit the table at the same time. This third situation was thought to be contradictory or non-intuitive for most of the students, because of focusing only on the differences in the mass (Kavanagh & Sneider, 2007), which was kept constant throughout the three situations. During these three POE cycles, students were expected to observe the pattern in the phenomenon that as the masses of the muffin cup piles (objects) increase, the gap in their falling times decreases.

### Epistemic Emotions

After each POE situation, as a part of the task, students were asked to self-report their experiences on situational epistemic emotions. Furthermore, epistemic emotions were measured at the beginning of the activity, as baseline measures, to use them as covariates in the subsequent analyses. Thus, the emotions were reported four times in total. The data collection on epistemic emotions was conducted following the guidelines of experience sampling method (ESM; Goetz et al., 2016). In collecting intensive, momentary ESM data, it is important to keep the questionnaire as short and easy to answer as possible. Thus, The Epistemically-Related Emotions Scale (EES; Pekrun et al., 2017) was modified to meet the needs of the present study: the single-item short scale of the EES was employed, in which a four-point Likert scale with the response categories from 1 = *not at all* to 4 = *very much* was used. Furthermore, only four epistemic emotions (out of seven in the original scale) were surveyed. In each questionnaire, students were asked: “How do you feel right now? Surprised/Curious/Confused/Bored”. The epistemic emotions of surprise, curiosity, and confusion were chosen for this study, because we expected especially these emotions to be present in this kind of a POE activity with contradictory and incongruous information. In turn, boredom was included in the questionnaire to represent a deactivating, negative epistemic emotion.

### Analyses

**Fig. 2:**
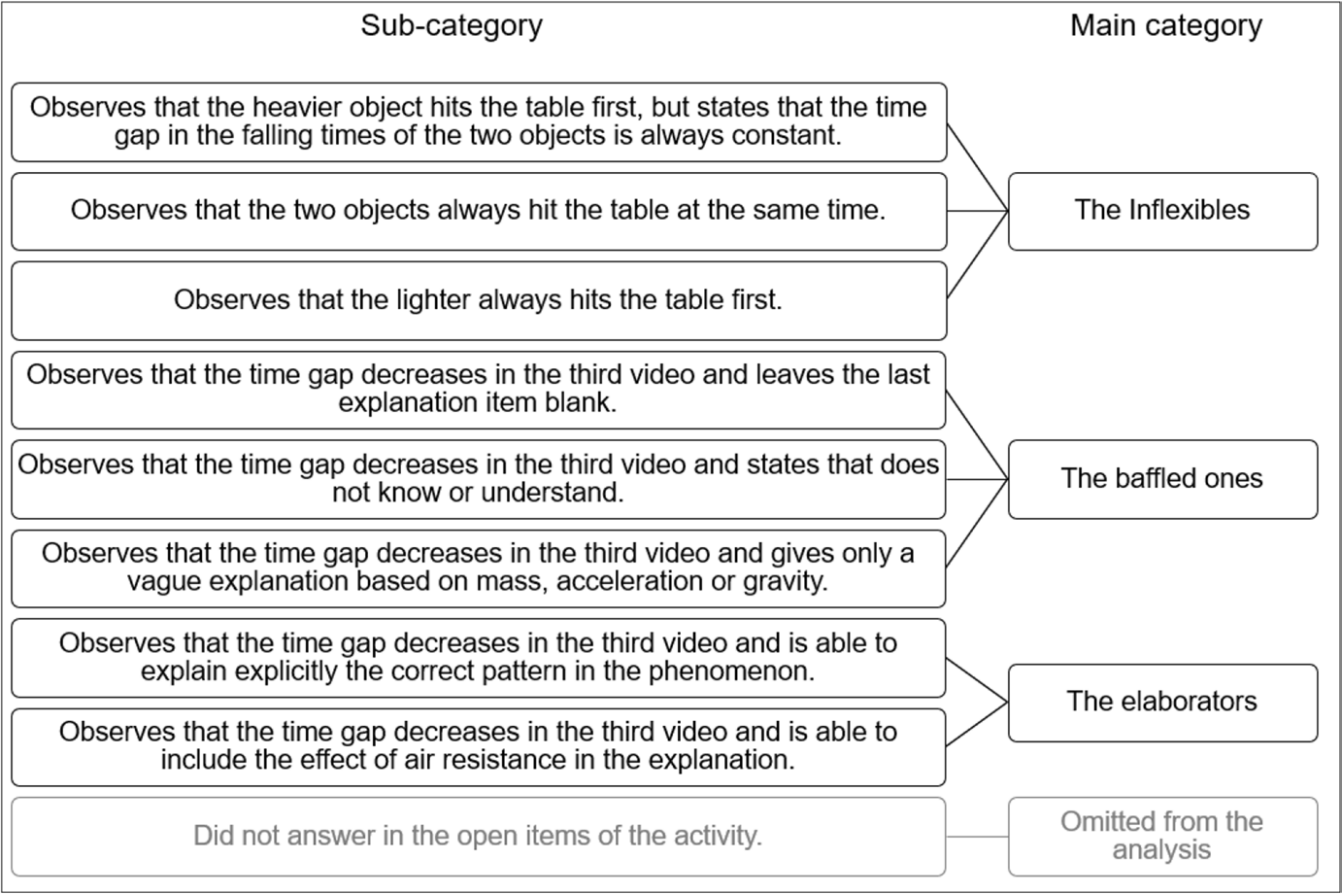
Sub-categories and main categories based on the inductive content analysis

To answer RQ1, students’ performance in sensemaking was evaluated based on their answers on the multiple choice and open answer POE items. Students’ individual answer patterns were analysed and categorised using qualitative and inductive content analysis (Elo & Kyngäs, 2008). According to Elo and Kyngäs (2008), a content analysis consists of three main phases: preparation, organising, and reporting. In the preparation phase, the overall answer pattern of an individual student was chosen to be the unit of the analysis. Also, the researchers became familiar with the data to make initial sense of the answer patterns. Based on the data, it was decided to consider following issues when analysing the data: (1) whether a student observes that the time gap in falling time is smaller in the last situation than in previous situations and (2) whether a student is able to organise his or her observations into scientifically meaningful patterns and thus to explain and make sense of the phenomenon. For example, Eberbach and Crowley (2017) emphasise the importance of organising phenomena into scientifically meaningful patterns during observations. In the present study, a scientifically meaningful pattern refers to the issue that as the masses of the objects increase, the time gap in their falling times decreases. During the organising phase, the data were organised in sub-categories and main categories (Fig. 2). First, the category descriptions were drafted, based on the initial understanding of the data, to describe the aspects of the answer patterns in the main categories. Second, two of the researchers analysed and categorised all the students’ answer patterns individually, working systematically according to the main category descriptions. Third, the answer patterns that the researchers had marked differently were reviewed together in respect to the evaluation criteria and the category descriptions. Fourth, the category descriptions were revised to address all the aspects of the data better, and the answer patterns with the different markings in the first round were categorised accordingly until a mutual consensus was reached. Four students were excluded from the analyses because they had not given any open answers, but only multiple choice answers. Thus, the answer patterns of 109 students were further analysed. If there was a discrepancy between multiple choice and open answers in some situation, the open answer was considered to be a primary answer, and the multiple choice answer was ignored. The final category descriptions are reported in the “Results” section.

To answer RQ2, students reported epistemic emotions were statistically analysed with Mplus 8.5 (Muthén & Muthén, 2017). Students’ longitudinal trajectories of epistemic emotions were examined using a conditional latent class growth analysis (LCGA) (Jung & Wickrama, 2008; Nagin, 1999), in which the baseline measures of epistemic emotions were used as covariates. Due to missing data, 109 cases were included in the analysis. LCGA allows for the identification of distinctive longitudinal answer patterns, i.e. trajectories, within a population. LCGA is a special type of growth mixture modelling (GMM) (Muthén, 2004) in which the within-group variation is fixed to zero. To decide the number of the latent classes, five criteria were used: the Vuong–Lo–Mendell–Rubin (VLMR) test of fit, Bayesian information criteria (BIC), Akaike information criteria (AIC), entropy values, and the clarity and interpretability of the classes. The best fitting model is considered to have a significant (< 0.05) VLMR *p*-value, low BIC and AIC values, a high entropy value, and to be theoretically consistent and interpretable.

Finally, the relationship between students’ performance in scientific sensemaking and their emotional trajectories (RQ3) was explored by a cross-tabulation of the answer pattern categories and emotional trajectory classes. A Pearson’s chi-square test was conducted using IBM SPSS Statistics 26.0.

## Results

### Students’ Performance in Making Sense of the Phenomenon

The inductive content analysis of the students’ responses showed that the individual answer pattern for the three-cycle POE activity could be categorised into three qualitatively distinctive main categories that illustrate the students’ different ability to make sense of the motion phenomena (examples of answer patterns of each category are given in Table 1):

1. The first group of students (*n* = 20; 18.3%), *The inflexibles*, were unable to observe that the gap in the objects’ falling times decreased in the course of the POE activity and thus were unable to make sense of the phenomenon. Most of the students in this group observed that the heavier object hit the table first, but the time gap in the falling times of the two objects was constant. In addition, some students in this group observed that the two objects always hit the table at the same time or that the lighter hit the table first.

**Table 1:** Examples of students’ answer patterns of the three categories demonstrating distinctive performances in scientific sensemaking

	Item	Student A	Student B	Student C
First situation	P1	*The heavier hits the table first*	*They hit the table at the same time*	*The heavier hits the table first*
	P2	Because the other object has bigger mass, so its air resistance is smaller	I don’t know; there was some reason for this, gravity?	The heavier object hits the ground first, because it has a stronger gravity
	O1	*The heavier hit the table first*	*The heavier hit the table first*	*The heavier hit the table first*
	E	[no answer]	Because the earth attracts the heavier object more due to gravity	The object that has a bigger mass has a greater falling speed
Second situation	P1	*The heavier hits the table first*	*The heavier hits the table first*	*The heavier hits the table first*
	P2	*Equal to the previous situation*	*Equal to the previous situation*	*Bigger than in the previous situation*
	P3	Because the difference in the masses of the objects does not change	Because their ratio is exactly the same as in previous one	The difference between the masses of the objects increases, so the time gap in their falling times should increase
	O1	*The heavier hit the table first*	*The heavier hit the table first*	*The heavier hit the table first*
	O2	*Equal to the previous situation*	*Equal to the previous situation*	*Smaller than in the previous situation*
	E	[no answer]	Because the earth attracts them in the same ratio as in the first situation	The objects hit the table almost at the same time. The increase of the masses of the objects caused a decrease in the time gap in their falling times
Third situation	P1	*The heavier hits the table first*	*The heavier hits the table first*	*They hit the table at the same time*
	P2	*Equal to the previous situation*	*Equal to the previous situation*	
	P3	Still the difference does not change	Because the situation is still the same	Based on previous videos, as the masses increase the time gap in their falling times decreases
	O1	*The heavier hit the table first*	*They hit the table at the same time*	*They hit the table at the same time*
	O2	*Equal to the previous situation*		
	E	[no answer]	I don’t know!	As the masses of the objects increase, the time gap in their falling times decreases

Note.

P1: Predict, what will happen (*multiple choice*)

P2: If you chose that the objects hit the table at different times, how does the situation differ from the previous situation? The time gap between object 1 and object 2 hitting the table is (*multiple choice*)

P3: Rationalise your choice. Why would this happen? (open answer)

O1: What did you observe? (*multiple choice*)

O2: If you observed that the objects hit the table at different times, how does the situation differ from the previous situation? The time gap between object 1 and object 2 hitting the table is (*multiple choice*)

E: Explain your observation. Why did this happen? (open answer)

Student A in Table 1 is an example of a student who considers that the heavier object always falls faster, but the time gap in their falling times must stay equal due to equal mass ratios. Thus, he or she is unable to observe that the time gap actually decreases and is practically non-existent in the third situation.

(2) The second group of students (*n* = 65; 59.6%), *The baffled ones*, observed that the time gap decreased in the third video: they stated either that the two objects hit the table at the same time or that the time gap in the falling times was smaller than in previous situations. However, the utterances in this category did not indicate the pattern in the phenomenon. Many of the students in this group left the last explanation item blank or stated that they did not know or understand. Those students, who did give an answer in the last item, were only able to give a vague explanation based on mass, acceleration, or gravity, such as “because the masses were big enough” or “because they have the same acceleration”.

Student B in Table 1 represents a student who indicates that situations 1 and 2 are similar: the heavier falls faster, and the time gap is equal. However, in situation 3, he or she observed that the objects fall at the same time but was unable to give any explanation for this.

(3) The third group of students (*n* = 24; 22.0%), *The elaborators*, also observed that the time gap decreased in the third video. Furthermore, they were able to observe and explain explicitly the correct pattern in the phenomenon: as the masses of the objects increase, the time gap in their falling times decreases. In addition, those students who were able to include the effect of air resistance in their explanations, thus being able to make scientific sense of the phenomenon, were included in this group.

Student C in Table 1 is an example of a student who can elaborate his or her answers based on observations. For example, in the second situation, he or she predicted that the time gap between the falling times of the objects would increase due to bigger masses. However, after making correct observations in consecutive situations, he or she could draft an explanation and explicitly state that as the masses increase the time gap decreases.

### Trajectories of Situational Epistemic Emotions

**Fig. 3:**
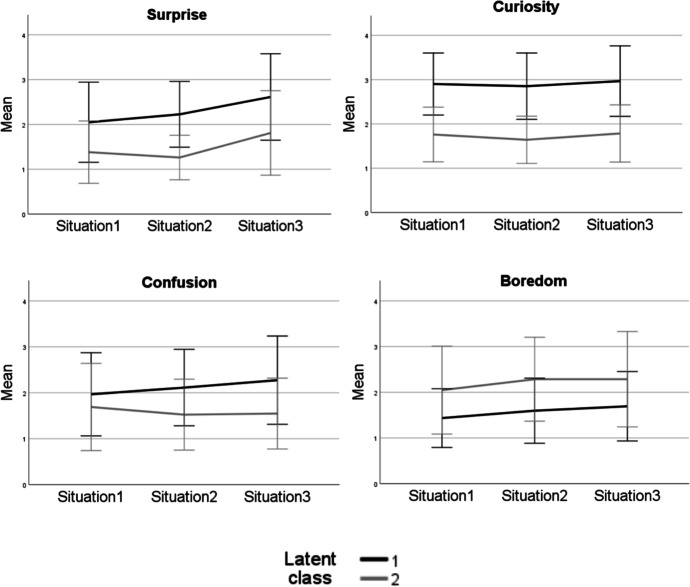
Means and standard deviations of the reported levels of epistemic emotions (surprise, curiosity, confusion, and boredom) in the two latent classes

The descriptive statistics of the situational epistemic emotions experienced in three situations of the POE activity are shown in Table 2. The LCGA was used to explore the latent emotional trajectories in the data, and a two-class solution was found to fit the data best (Table 3). The first latent class includes 63 (57.8%) students and is characterised by high levels of curiosity in addition to increasing levels of surprise and confusion. The second latent class includes 46 (42.2%) students and, in contrast, is characterised by high levels of boredom (Fig. 3). Even though the students in the second class also show an increase in the level of surprise in the third situation, this is not related to an increase in curiosity or confusion.

**Table 2:** Descriptive statistics of epistemic emotions of the two latent emotional trajectory classes

		Total			Latent class 1			Latent class 2		
Situation	Emotion	*n*	*Mean*	*S.D*	*n*	*Mean*	*S.D*	*n*	*Mean*	*S.D*
1	Surprised	110	1.76	0.88	62	2.05	0.90	46	1.39	0.71
	Curious	111	2.40	0.89	62	2.90	0.69	46	1.72	0.62
	Confused	109	1.83	0.92	62	1.97	0.91	45	1.67	0.93
	Bored	109	1.69	0.84	62	1.44	0.64	45	2.04	0.95
2	Surprised	108	1.85	0.81	62	2.23	0.73	43	1.28	0.50
	Curious	108	2.36	0.89	62	2.85	0.74	43	1.65	0.53
	Confused	108	1.88	0.85	62	2.11	0.83	43	1.53	0.77
	Bored	107	1.86	0.86	62	1.60	0.71	43	2.26	0.93
3	Surprised	108	2.28	1.03	63	2.62	0.96	42	1.81	0.94
	Curious	107	2.50	0.95	62	2.98	0.80	42	1.79	0.65
	Confused	108	1.98	0.95	63	2.29	0.96	43	1.56	0.77
	Bored	107	1.93	0.93	63	1.68	0.76	42	2.29	1.04

**Table 3:** Fit indices for the compared conditional LCGA models

Model	VLMR *p*-value	BIC	AIC	Entropy
1-class		4603.03	4526.91	
2-class	0.016	3244.57	3155.76	0.85
3-class	0.134	3190.78	3066.98	0.89

### Performance in Scientific Sensemaking and Emotional Trajectories

The descriptive statistics of the epistemic emotions in the three distinctive sensemaking categories are shown in Table 4. According to a chi-square test of independence, there is a significant relationship between students’ performance in scientific sensemaking evaluated by a qualitative content analysis and students’ emotional trajectories found through LCGA, *X2*(2, *N* = 106) = 10.78, *p* = 0.005. *The inflexibles* are more likely to belong to the latent class characterised by high levels of boredom, whereas *The baffled ones* and *The elaborators* are more likely to belong to the latent class with high levels of curiosity, surprise, and confusion. There was no significant difference between *The baffled ones* and *The elaborators* in terms of their belonging to the emotional trajectory classes (Table 5).

**Table 4:** Descriptive statistics of epistemic emotions for the three sensemaking categories

		1. The inflexibles			2. The baffled ones			3. The elaborators		
Situation	Emotion	*n*	*Mean*	*S.D*	*n*	*Mean*	*S.D*	*n*	*Mean*	*S.D*
1	Surprised	20	1.45	0.61	64	1.80	0.89	23	1.91	1.00
	Curious	20	2.20	1.06	64	2.41	0.90	24	2.54	0.72
	Confused	20	1.55	0.76	63	1.87	0.98	23	1.96	0.88
	Bored	20	1.60	0.75	63	1.78	0.92	23	1.57	0.66
2	Surprised	19	1.68	0.82	62	1.85	0.83	24	2.00	0.78
	Curious	19	2.16	1.07	62	2.31	0.86	24	2.67	0.82
	Confused	19	1.63	0.76	62	1.92	0.84	24	2.00	0.98
	Bored	19	1.95	0.91	62	1.77	0.90	23	1.96	0.77
3	Surprised	18	1.28	0.58	63	2.56	1.00	24	2.38	0.97
	Curious	18	2.06	1.11	62	2.58	0.95	24	2.63	0.77
	Confused	18	1.39	0.61	64	2.19	0.97	23	2.00	0.91
	Bored	18	2.22	1.06	63	1.90	0.98	23	1.70	0.64

**Table 5:** The relation (Pearson’s chi-square test) between students’ epistemic emotions and performance in sensemaking

Emotional trajectory categories	1. The inflexibles *n* = 19 *n* (%)	2. The baffled ones *n* = 64 *n* (%)	3. The elaborators *n* = 23 *n* (%)
1. Curious, increasing surprise and confusion	5 (26.3)^A^	40 (62.5)^B^	17 (73.9)^B^
2. Bored	14 (73.7)^A^	24 (37.5)^B^	6 (26.1)^B^

Note. The subscript letters ^A^ and ^B^ denote the categories whose column proportions differ significantly from each other at the *p* < 0.05 level

## Discussion

In this study, we investigated upper secondary students’ performance in making sense of the scientific phenomenon and their emotional trajectories during a POE activity and how these performances and trajectories are related. The results of the study are based on the qualitative data on students’ predictions, observations and explanations, and quantitative data on students’ reported levels of situational epistemic emotions. The findings provide novel perspectives for science educators and researchers on how students’ emotions and their learning processes are intertwined. The findings suggest that both emotions and cognition, affecting one another, play a role in scientific sensemaking. The details of examples are discussed in the following sections.

### Role of Relevant Observations in Scientific Sensemaking

In our data, most of the students were categorised as *The baffled ones*, who were able to notice that the time gap in the falling times of the two objects decreased in the last situation but were unable to explain it in depth. Furthermore, about one-fifth of the students were categorised as *The elaborators*, who were able to observe the pattern in the phenomenon or link their observations to their previous knowledge on scientific concepts (e.g. air resistance), thus showing good performance in scientific sensemaking. The reason for the prevalence of students, who were only able to notice and not give a theoretical interpretation, is probably because students’ prior knowledge from lower secondary school was not sufficient to make further scientific inferences. As other studies have also suggested, prior knowledge is a prerequisite for making relevant scientific observations (Hodson, 1996; Kohlhauf et al., 2011; Pinch, 1985). As indicated in the “Analyses” and “Results” sections of this paper, a variety of sub-categories were included in each main category, showing that there was also a diversity of students’ answers within main categories. Whereas *The inflexibles* differ relatively significantly from the other two categories, the difference between *The baffled ones* and *The elaborators* is more subtle. Both *The baffled ones* and *The elaborators* were able to more or less make the expected observations. The two categories were distinguished only based on the students’ different ability, or readiness, to explain their observations. Indeed, it should be noted that the computer-based POE task used in this study was very low-stakes, and time available for answering the items of the tasks was relatively short. So, if a student did not have the explanation readily in his or her mind, it might have been tempting to just leave the last few items empty instead of trying to construct an explanation with great cognitive effort.

In the data, almost one-fifth of the students, *The inflexibles*, did not indicate the time gap getting smaller, or they indicated that the objects always hit the table at the same time, regardless of their mass. The open answers (i.e. the rationales for predictions and the explanations) underline the finding that quite several students were very fixed in their preconceptions and thus had problems making observations that were contrary to their preconceptions. This is in line with Hodson’s (1996) argument that students’ intuitive views can constitute a barrier to observation. Our findings also support previous studies showing that it is common for students to have difficulties in making scientific observations (Haigh et al., 2012; Remmen & Frøyland, 2020). Furthermore, the findings may indicate that students often lack the ability to link their observations with the knowledge they already have in science.

Considering these findings in the context of science teaching and learning, teachers should not assume that students can always make the right observations when watching demonstrations or during inquiry-based learning. Instead, teachers should scaffold students in making observations by, for example, breaking up the practice of scientific observation into smaller fragments or posing relevant questions (Ahtee et al., 2011; Smith & Reiser, 2005). Also, based on our findings and supported by the literature (e.g. Haigh et al., 2012; Remmen & Frøyland, 2020), we argue that making observations should be practised more often in science education. However, as previous research has shown, teachers may also have difficulties in guiding observations (Ahtee et al., 2011). Thus, emphasis in teacher training should be placed on providing basic tools for teachers on how to guide students during observational practices. Teachers should be able to scaffold students in focusing their attention on the relevant questions at the relevant time and thus help students to notice such interrelations or trajectories that are contrary to one’s preconceptions.

### Trajectories of Situational Epistemic Emotions During Scientific Sensemaking

The POE activity used in this study was designed with the intention that as the activity proceeds, the contents of the videos become contradictory or incongruous for the students, thus arousing surprise in students and then curiosity and/or confusion. Based on the LCGA, more than half of the students had the somewhat emotional experience that was expected: they felt more surprised and confused towards the end of the task, but their levels of curiosity were constantly high. This finding is also in line with previous research suggesting positive emotions occurring during POE activities (Bellocchi et al., 2014). However, based on our data, we cannot say that surprise aroused by incongruous information led to curiosity, as previously suggested by Vogl and colleagues (2020), for example. Instead, our data support the view that those students who are already curious during the first situation of the POE activity are more likely to experience surprise and confusion at later stages. This emotional trajectory with relatively high levels of surprise, curiosity, and confusion can be considered favourable in terms of the positive learning outcomes these emotions are often linked to (Theobald & Brod 2021; Vilhunen et al., 2022; Vogl et al., 2020; Wu et al., 2018). The occurrence of surprise, curiosity, and confusion in the learning situations with incongruous information or high cognitive demands has also been shown in previous studies (Chiu et al., 2019; Haigh et al., 2012; Vilhunen et al., 2021; Vogl et al., 2020).

Furthermore, almost half of the students in our data were notably bored throughout the learning task. This finding is also in line with the view that the emotions experienced at the beginning of a learning task have an influence on the later experiences. Even though the group of bored students experienced increasing levels of surprise in the third situation, this contradictory situation had no effect on their experiences of curiosity or confusion, which would be important in terms of using deep-processing learning strategies (Muis et al., 2015). This emotional trajectory with relatively high levels of boredom can be considered to be undesirable, due to the negative associations that boredom has with many learning-related factors (Pekrun et al., 2014; Tze et al., 2016; Vilhunen et al., 2022). One reason for this high occurrence of boredom might be that most of our data were gathered during the 2020–2021 semester, when distance teaching was common in Finnish upper secondary schools due to COVID-19 pandemic. Thus, even if the students were in contact teaching when participating in data gathering, any computer-based learning task may have aroused boredom in them, due to excessive amount of online instruction during the semester.

### Students’ Observations and Their Emotional Trajectories Are Intertwined

Our results show that the levels of epistemic emotions vary significantly in relation to students’ ability to make relevant observations. Based on a cross-tabulation, we found that those students who have emotional trajectories with high levels of curiosity and increasing levels of surprise and confusion are able to make better sense of the phenomenon than students with high levels of boredom. This implies that boredom may have an inhibiting effect on observing and developing understanding, as also suggested in previous research (Darner, 2019; Muis et al., 2015; Tze et al., 2016; Vilhunen et al. 2022). Correspondingly, curiosity, confusion, and surprise may foster these cognitive processes and engagement in sensemaking. Thus, the findings corroborate those of previous studies (e.g. Jacobson et al., 2021; Theobald & Brod, 2021; Watkins et al., 2018; Wu et al., 2018). Especially curiosity has a role in critical thinking, namely in the ability to apply previous knowledge to new situations and in the evaluation of information (Muis et al., 2015). These deep-processing learning strategies are also central in making scientific observations. In turn, boredom, as a negative deactivating emotion, is related to impairment of any learning strategies, which in our case appears as an inability or unwillingness to make scientifically relevant observations.

Even though confusion is a negative emotion based on its valence, in terms of learning, it can be considered to be a positive emotion. Based on our findings, confusion often appears with the positive emotions of curiosity and surprise. This is in line with previous research suggesting a positive relation between confusion and learning (Muis et al., 2015). However, some researchers consider confusion to be an emotion that can detract from learning (Jacobson et al., 2022; Schneider et al., 2016). Our results, instead, indicate that situational confusion acts more typically as a learning enhancer. Overall, the findings of the present study corroborate the assumption that emotions interrelate with scientific sensemaking and reasoning (Fischer et al., 2014; Theobald & Brod, 2021; Wickman et al., 2022).

### Limitations of the Study

We acknowledge that situational emotions can be influenced by things such as moods or other personal dispositions to experience certain types of emotions (Shuman & Scherer, 2014). In addition, it is generally assumed that prior knowledge and preconceptions have an effect on the observations one makes (Hodson, 1996; Kohlhauf et al., 2011). However, in this study, we did not employ a comprehensive background questionnaire but instead only collected baseline measures for the epistemic emotions. By considering affective and cognitive (especially prior knowledge) background variables in future studies, it would be possible to explore in more detail what actually takes place in the situation itself and what is brought to the learning situation by a student, both affectively and cognitively.

This study was conducted in a highly controlled, laboratory-like learning environment and focused solely on the epistemic antecedents of students’ emotions. However, science lessons typically consist of diverse activities and events, in which emotions can be aroused by variety of different factors, such as achievement activities, topics being studied, or social interactions (e.g. Davis & Bellocchi, 2018; Pekrun et al., 2018). These factors, while acknowledged to play a significant role in science learning, were beyond the scope of this study. Furthermore, due to the convenience sampling approach employed in this study, caution with generalisations of the findings is warranted.

### Implications for Practice and Research

Based on the findings from this study, we argue that making sense of scientific phenomena, and especially making relevant observations, is difficult for many upper secondary students and thus should be practised as a fundamental part of science education. Observing often involves critical thinking and letting go of one’s preconceptions. These cognitive discrepancies may again cause negative emotions which are, however, pertinent to science learning (Chiu et al., 2019; Jaber & Hammer, 2016; Radoff et al., 2019). Thus, teachers should scaffold students through these unpleasant emotions instead of trying to avoid such learning situations in which confusion may occur. Furthermore, the results suggest that bored students can be unwilling or unable to make scientific observations and can be fixed in their preconceptions. Thus, engaging these bored students to curiously observe and test predictions is an important mission for curriculum designers and teachers in practice. Overall, our findings underline the importance of emotions in educational settings. Thus, future studies are needed to clarify the complexity of the interplay between cognitive and affective factors in learning situations.

## Supplementary Information

Below is the link to the electronic supplementary material.

Supplementary file1 (DOCX 217 kb)
