# Supplementary material for: Epistemic Emotions and Observations Are Intertwined in Scientific Sensemaking: A Study among Upper Secondary Physics Students
Source: Int J Sci Math Educ. 2022 Sep 5;21(5):1545–66. doi: 10.1007/s10763-022-10310-5 (PMC9442553; doi:10.1007/s10763-022-10310-5)
Supplement: Supplementary file 1 — Supplementary file1 (DOCX 217 kb) [file 10763_2022_10310_MOESM1_ESM.docx]

**Supplemental Material**

*The computer-based POE activity*

| Explaining motion phenomena  Using this form, the competence of explaining motion phenomena and emotions related to explaining is explored. The tasks of the form may feel difficult, because in the lower secondary school the topic has been covered only briefly. However, try to answer each question carefully. | |
| --- | --- |
| How do you feel right now? Answer the following items (1 Not at all … 4 Very much)  Surprised  Curious  Confused  Bored | |
| Situation 1:  Two objects fall, of which one has a double mass  Object 1 = 2 muffin cups; object 2 = 1 muffin cup  Predict which of the following happens (multiple choice answer)   - the heavier hits the table first - the lighter hits the table first - they hit the table at the same time   Explain your choice. Why would this happen?  (open answer) | Prediction  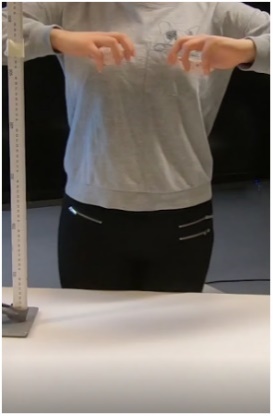 |
| Watch the video twice. Observe the falling objects. Was your prediction realised?  What did you observe? (multiple choice answer)   - the heavier hit the table first - the lighter hit the table first - they hit the table at the same time   Explain your observation. Why did this happen? (open answer)  How do you feel right now? Answer the following items (1 Not at all … 4 Very much)  Surprised  Curious  Confused  Bored | Observation  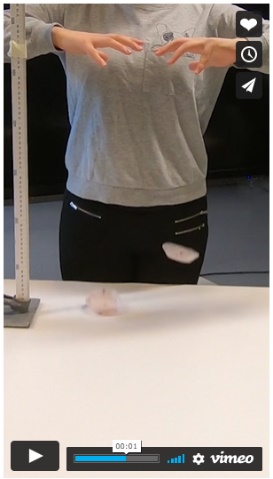 |
| Situation 2:  Two objects fall, of which one has a double mass  Object 1 = 4 muffin cups; object 2 = 2 muffin cups  Predict which of the following happens (multiple choice answer)   - the heavier hits the table first - the lighter hits the table first - they hit the table at the same time   If you chose that the objects hit the table at different times, how does the situation 2 differ from situation 1? The time gap between object 1 and object 2 hitting the table is (multiple choice answer)   - smaller than in situation 1 - bigger than in situation 1 - equal to situation 1   Explain your choice. Why would this happen?  (open answer) | Prediction  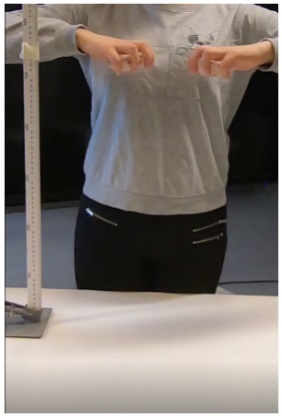 |
| Watch the video twice. Observe the falling objects. Was your prediction realised?  What did you observe? (multiple choice answer)   - the heavier hit the table first - the lighter hit the table first - they hit the table at the same time   If you observed that the objects hit the table at different times, how does situation 2 differ from situation 1? The time gap between object 1 and object 2 hitting the table is (multiple choice answer)   - smaller than in situation 1 - bigger than in situation 1 - equal to situation 1   Explain your observation. Why did this happen? (open answer)  How do you feel right now? Answer the following items (1 Not at all … 4 Very much)  Surprised  Curious  Confused  Bored | Observation  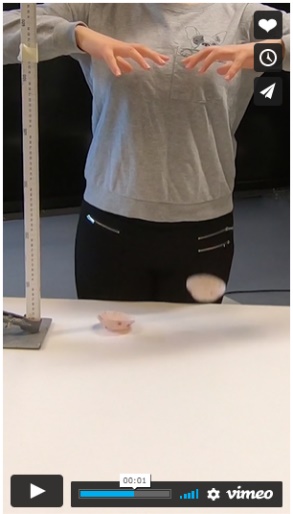 |
| Situation 3:  Two objects fall, of which one has a double mass  Object 1 = 32 muffin cups; object 2 = 16 muffin cups  Predict which of the following happens (multiple choice answer)   - the heavier hits the table first - the lighter hits the table first - they hit the table at the same time   If you chose that the objects hit the table at different times, how does situation 3 differ from situation 2? The time gap between object 1 and object 2 hitting the table is (multiple choice answer)   - smaller than in situation 2 - bigger than in situation 2 - equal to situation 2   Explain your choice. Why would this happen?  (open answer) | Prediction  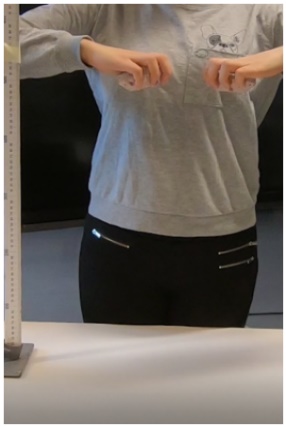 |
| Watch the video twice. Observe the falling objects. Was your prediction realised?  What did you observe? (multiple choice answer)   - the heavier hit the table first - the lighter hit the table first - they hit the table at the same time   If you observed that the objects hit the table at different times, how does situation 3 differ from situation 2? The time gap between object 1 and object 2 hitting the table is (multiple choice answer)   - smaller than in situation 2 - bigger than in situation 2 - equal to situation 2   Explain your observation. Why did this happen? (open answer)  How do you feel right now? Answer the following items (1 Not at all … 4 Very much)  Surprised  Curious  Confused  Bored | Observation  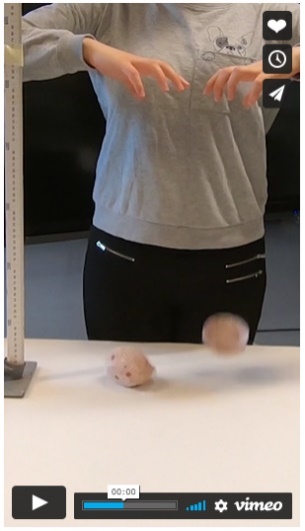 |
